# WORK-RELATED VIOLENCE OR HARASSMENT AND SICKNESS ABSENCE DUE TO COMMON MENTAL DISORDERS: A PROSPECTIVE TWIN STUDY

**DOI:** 10.1093/geroni/igad104.3699

**Published:** 2023-12-21

**Authors:** Linda Magnusson Hanson, Maria Wijkander, Jurgita Narusyte, Iman Alaie, Petra Lindfors, Tianwei Xu, Pia Svedberg

**Affiliations:** Stockholm University, Stockholm, Stockholms Lan, Sweden; Stockholm University, Stockholm, Stockholms Lan, Sweden; Karolinska Institutet, Stockholm, Stockholms Lan, Sweden; Karolinska Institutet, Stockholm, Stockholms Lan, Sweden; Stockholm University, Stockholm, Stockholms Lan, Sweden; Stockholm University, Stockholm, Stockholms Lan, Sweden; Karolinska Institutet, Stockholm, Stockholms Lan, Sweden

## Abstract

The aim of the present study was to investigate the association between exposure to unacceptable behaviors and practices or threats thereof in working life that result in or aim to result in physical, psychological, sexual or economic harm, such as violence and harassment including unwelcome conduct based on e.g. sex or gender (gender-based harassment/discrimination), and future sickness absence due to common mental disorders among Swedish twins. The study included 8795 twins, from the Swedish Study of Twin Adults: Genes and Environment, asked to report exposure to work-related violence or threats of violence and harassment or bullying and whom were followed-up for certified sickness absence (min 12 years of follow-up). Standard logistic regression indicated that exposure increased the odds of sickness absence due to common mental disorders (adjusted OR 2.11; 95% CI 1.52-2.95 for violence/threats, adjusted OR 1.52; 95% CI 1.10-2.11 for harassment/bullying). A co-twin control analyses based on conditional logistic regression restricted to twin pairs discordant for exposure, in which a co-twin control was used as reference, however, resulted in attenuated ORs (2.0; 0.79-5.07 and 1.56; 0.66-3.66, respectively). This indicates that the relationships could be at least partially confounded by familial factors, such as genetics and shared environment. These results suggests that more work is needed to clarify whether there is a causal association between these types of unacceptable behaviors/practices and mental health outcomes considering familial/genetic factors, and whether prevention of such behaviors/practices can keep individuals increasingly healthy, active on the labor market and promote healthy aging.

